# Attenuating nicotine’s effects with high affinity human anti-nicotine monoclonal antibodies

**DOI:** 10.1371/journal.pone.0254247

**Published:** 2021-07-30

**Authors:** Michael D. Raleigh, Nicola Beltraminelli, Stephanie Fallot, Mark G. LeSage, Amy Saykao, Paul R. Pentel, Steve Fuller, Thomas Thisted, Zuzanna Biesova, Stephen Horrigan, Darryl Sampey, Bin Zhou, Matthew W. Kalnik

**Affiliations:** 1 Hennepin Healthcare Research Institute, Minneapolis, Minnesota, United States of America; 2 Department of Pharmacology, University of Minnesota, Minneapolis, Minnesota, United States of America; 3 Blink Biomedical, Marseille, France; 4 Nic•mAb Strategic Alliance, San Diego, California, United States of America; 5 Antidote Therapeutics, Inc., Woodbine, Maryland, United States of America; 6 Noble Life Sciences, Woodbine, Maryland, United States of America; 7 Biofactura, Inc., Frederick, Maryland, United States of America; 8 The Scripps Research Institute, La Jolla, California, United States of America; University of California San Diego, UNITED STATES

## Abstract

Use of nicotine-specific monoclonal antibodies (mAbs) to sequester and reduce nicotine distribution to brain has been proposed as a therapeutic approach to treat nicotine addiction (the basis of tobacco use disorder). A series of monoclonal antibodies with high affinity for nicotine (nic•mAbs) was isolated from B-cells of vaccinated smokers. Genes encoding 32 unique nicotine binding antibodies were cloned, and the mAbs expressed and tested by surface plasmon resonance to determine their affinity for S-(–)-nicotine. The highest affinity nic•mAbs had binding affinity constants (*K*_D_) between 5 and 67 nM. The 4 highest affinity nic•mAbs were selected to undergo additional secondary screening for antigen-specificity, protein properties (including aggregation and stability), and functional *in vivo* studies to evaluate their capacity for reducing nicotine distribution to brain in rats. The 2 most potent nic•mAbs in single-dose nicotine pharmacokinetic experiments were further tested in a dose-response *in vivo* study. The most potent lead, ATI-1013, was selected as the lead candidate based on the results of these studies. Pretreatment with 40 and 80 mg/kg ATI-1013 reduced brain nicotine levels by 56 and 95%, respectively, in a repeated nicotine dosing experiment simulating very heavy smoking. Nicotine self-administration was also significantly reduced in rats treated with ATI-1013. A pilot rat 30-day repeat-dose toxicology study (4x200mg/kg ATI-1013) in the presence of nicotine indicated no drug-related safety concerns. These data provide evidence that ATI-1013 could be a potential therapy for the treatment of nicotine addiction.

## Introduction

Tobacco use is the leading preventable cause of illness and death in the world today [1]. Over 36 million Americans smoke tobacco [2]. Sixteen million live with a smoking-attributable disease and yet 30–40% of these individuals continue to smoke [3]. Cigarette smoking is responsible for over 400,000 deaths (1 out of every 5) in the United States each year [3]. Most smokers are aware of the health consequences and want to quit, but have difficulty doing so. Only 3–5% of smokers who quit on their own are successful in maintaining long-term abstinence [4]. Approved pharmacotherapies when coupled with behavioral counseling can lead to long-term abstinence rates up to 20–25% compared to 9% receiving counseling alone [5]. Given this modest long-term efficacy, a significant unmet medical need exists for more effective and complementary approaches to treat nicotine addiction.

Sequestration of drugs of abuse using drug-specific antibodies to slow and reduce drug distribution to brain is a novel therapeutic approach being investigated to treat drug addiction [6,7]. The nicotine-blocking effects of nicotine-specific monoclonal antibodies (nic•mAbs) have been studied in rodents, with up to ~80% reduction in nicotine brain levels observed within a few minutes of intravenous (i.v.) dosing of nicotine [8,9]. Dose-dependent and affinity-dependent responses have also been demonstrated [8,9]. Nic•mAbs are particularly effective in reducing the early distribution of nicotine to brain, which is important because the greatest reinforcing and subjective effects occur within the first few minutes of smoking [10]. Since nicotine in the blood is the target of the antibody, rather than the nicotinic acetylcholine receptors in the brain, the antibody circumvents the central nervous system, which may help to avoid the neuropsychiatric adverse effects that can accompany other pharmacotherapies [11,12].

Pre-clinical and clinical research has been conducted on nicotine conjugate vaccines that elicit polyclonal anti-nicotine antibodies [13–16] which helps to inform the optimization and development of nic•mAbs for smoking cessation [17]. Two large Phase III studies did not demonstrate effectiveness of a nicotine vaccine as an aid to smoking cessation in the intent-to-treat (ITT) population [18,19]. However, long-term abstinence rates were effectively doubled in individuals who attained the highest anti-nicotine antibody titers (top third) compared to placebo and the low titer groups for two independently developed vaccines [13,14]. Brain imaging studies undertaken for both vaccines demonstrated reduction in brain nicotine levels by 15–23% on average in the high titer group [20,21]. Consistent with these imaging results, a physiologic-based pharmacokinetic model [22] predicts maximal lowering of brain nicotine of only 15% when modeling antibody levels and affinities observed in the vaccine clinical studies (44 μg/mL [14], *K*_D_ = 37 nM [23]).

The lack of a robust clinical effect has been hypothesized to arise from a variety of factors: [7,16,17,24,25] (a) the levels of vaccine-elicited Ab’s were too low on average to produce a therapeutic effect across the entire treatment group, (b) the Ab titers that were produced varied by several orders of magnitude between smokers, (c) the Ab’s had insufficient affinity for binding nicotine as the vaccines elicited a mixture of polyclonal Ab’s with a range of affinities for both stereoisomers of nicotine [25] (the S-(–)-nicotine enantiomer is almost exclusively found in tobacco (>99%) and is significantly more psychoactive than the R-(+)-nicotine enantiomer [26]), and (d) multiple vaccinations took 3–4 months to achieve maximal antibody levels which may have had an adverse influence on the quitting process in smokers as delaying attempts at quitting have been correlated with less success among smokers actively trying to quit [27].

Despite these factors, evidence of improved smoking cessation and abstinence rates relative to placebo were observed in treated smokers who attained high antibody titers. Based on a pre-specified stratification prior to trial unblinding, the top-third of vaccine responders by antibody-AUC in the randomized, placebo-controlled Phase II [13,14] and Phase III [13,14,16,19] trials, showed an approximate 2-fold improvement in long-term abstinence rates. The geometric mean titers reached by this high-responder group at the target quit date (TQD) in Phase III approached 100 μg/mL [16,28]. These clinical results suggest that efficacy likely requires achieving nicotine-specific antibody levels in serum of at least 100 μg/mL. In order to achieve high serum levels of uniform, high-affinity anti-nicotine antibodies, we set out to discover and optimize fully-human, enantiospecific, anti-nicotine monoclonal antibodies as a potential smoking cessation treatment.

Here we describe our efforts isolating and characterizing fully human nic•mAbs with high affinity for free S-(–)-nicotine from individuals previously administered an investigational nicotine vaccine. Single B-cells from pools of peripheral blood mononuclear cells (PBMCs) isolated from these subjects were screened for their ability to produce antibodies which bound with high efficiency to a nicotine conjugate as measured by an enzyme-linked immunosorbant assay (ELISA) [29]. After cloning their corresponding heavy and light chain genes, 32 individual nic•mAbs were produced in small scale by transient transfection, purified, and subsequently ranked for their binding affinity to free S-(–)-nicotine in solution using surface plasmon resonance (SPR) biosensor analysis. The focus of this report is the characterization of the most potent and highly specific nic•mAbs isolated, the *in vivo* evaluation of the highest affinity mAbs for their ability to block nicotine distribution to brain and assessment of efficacy in animal models of addiction.

## Methods

### Ethics statement

The clinical blood collection study (ATI-1501) was evaluated and approved by MaGil Institutional Review Board (under contract to Antidote Therapeutics, Inc.) and the National Institutes of Health and conducted in accordance with NIH Human Subjects Research Policy and Guidelines. Animal studies were performed in accordance with the Guide for the Care and Use of Laboratory Animals of the National Institutes of Health. Animal protocols were approved by the Hennepin Healthcare Research Institute and Noble Life Sciences (under contract to Antidote Therapeutics, Inc.) Institutional Animal Care and Use Committees. Surgery was performed under ketamine (75 mg/kg) and dexmedetomidine (0.05 mg/kg) or under 3% isoflurane anesthesia, animals were euthanized by CO_2_ or 5% isoflurane inhalation using AAALAC approved chambers, and all efforts were made to minimize suffering.

#### Animals

Studies used Sprague Dawley (SD) or Holtzman Sprague Dawley (HSD) rats (Envigo, Madison, WI or Charles River, PA) depending on availability and were double housed under a 12/12-h standard light/dark cycle, free-fed, and testing occurred during the light phase. In self-administration studies SD rats were individually housed under a 12/12-h light/dark cycle, food was restricted to 18–20 g/day, and self-administration sessions occurred during the dark phase of the light cycle. Food restriction was imposed for self-administration rats because it is healthier than free-feeding for rats during long-term studies and can facilitate acquisition of drug self-administration [30,31]. Self-administration occurred during the dark phase because this is typical for studies using short-access sessions and it models the time of day when smoking (i.e., nicotine self-administration) occurs in humans [26,31]. Male and female rats were used in different experiments to increase heterogeneity. Female rats were used during initial studies when availability of monoclonal antibody was limited (Experiments 1 and 3). Both male and female rats were used for critical experiments (Experiments 2, 6 and 7). Experiment-specific details are provided below.

#### Donation of PBMCs from previously vaccinated individuals

A clinical sample collection study designed to collect blood from subjects who had received repeated injections of a nicotine conjugate vaccine (3’AM-Nic-rEPA, consisting of the racemic hapten 3’-aminomethyl-nicotine (3’AM-Nic), the structure of which is published elsewhere [32], linked to the amino acid side-chain and terminal amino groups of Pseudomonas aeruginosa exoprotein A (rEPA) via a succinyl linker) for smoking cessation in the past 10 years was conducted by Optimal Research (Rockville, MD and surrounding areas). Subjects (male and female) between 21 and 75 years old who were immunocompetent, had hematological test results that were in the normal range, and in good general physical and mental health, were recruited as specified in the study protocol (ATI-1501). ATI-1501 is a blood sample collection clinical protocol that included routine procedures similar to standard blood donation practices as follows: After screening for eligibility and provision of informed consent, 450-mL blood samples were obtained from each of three participants and processed to isolate PBMCs. Personal identifiable information was inaccessible to all individuals who were not directly conducting the study.

#### Nic•mAb lead generation

IgM+/IgD+ cells were depleted from the PBMC pools collected under protocol ATI-1501 to enrich the population of IgG+ B-cells. After seeding 200–250 IgG+ B-cells per well in a 96-well plate, the B-cells were transformed by infection with the Epstein-Barr virus and co-cultured in the presence of CD40L-expressing feeder cells for amplification. After 2 weeks, the culture supernatants were screened for the presence of secreted anti-nicotine antibodies in the ELISA protocol outlined below using racemic 3’-aminomethyl-nicotine-polyglutamic acid (3’AM-Nic-pGlu) as the coating antigen for this initial screening, indicating that at least one B-cell within the pool in the well was producing anti-nicotine antibodies. After identification of a positive B-cell pool, the corresponding cells were deposited on an ISAAC^™^ (ImmunoSpot Array Assay on a Chip) microarray [33] coated with racemic 3’AM-Nic-pGlu conjugate at 10 μg/ml. The array consists of wells designed to accommodate a single lymphocyte in each well. After a 3 h incubation, cells secreting nicotine-specific antibodies were identified by staining with a fluorescently labelled secondary anti-IgG antibody (anti-human IgG(Fab’)_2_-Cy3), which stains around the wells in which a B lymphocyte had secreted a nicotine conjugate-specific IgG. Single antibody-positive cells were retrieved by micromanipulation, and the DNA sequences for the IgG light- and heavy-chains in those cells were isolated by RT-PCR and cloned into expression vectors to produce recombinant IgG1 candidates via transient transfection of CHO-S cells and purification by Protein A affinity chromatography. The purified mAbs were then dialyzed into phosphate buffered saline (PBS; 20 mM phosphate, 150 mM NaCl, pH 7.4), and concentration determined by A_280_ using an estimated extinction coefficient of 1.4 based on the protein sequence. In summary, 54 million total B-cells were screened, 146 B-cell pools were identified as 3’AM-Nic-pGlu binders in ELISA and single cells could be retrieved for 64 hits. These hits were then further qualified by competition ELISA to S-(–)-nicotine while using 3’AM-S-(–)-Nic-pGlu conjugate as the plating antigen in order to focus only on mAbs specific to S-(–)-nicotine and eliminate those specific to pGlu or an epitope covering the linker, and counter-screened in competition ELISA with S-(–)-cotinine, also using 3’AM-S-(–)-Nic-pGlu conjugate as the plating antigen. Finally, 32 novel antibodies were progressed to the production/purification phase.

#### Conjugate binding ELISA

The conjugate used in the ELISA assay (except for the initial screening described above) was an enantiopure form of the racemic hapten used in the original vaccine, since we were interested in monitoring the binding to the S-(–) form of nicotine exclusively. Briefly, ELISA plates were coated with 1 ng/ml of 3’AM-S-(–)-Nic-pGlu conjugate diluted in coating buffer (0.1M sodium phosphate) overnight at 4°C. Plates were blocked for 1 h at room temperature with blocking and dilution buffer (1% Non-Fat Dry Milk (NFDM) in 1X PBS). Dilutions of sample (culture supernatant or purified mAbs) in PBS + 1% NFDM were added to the blocked wells and incubated for 90 min at 37°C. Plates were washed 5 times with washing buffer (1X PBS + 0.1% Tween 20) and incubated for 60 min at 37°C with Goat α-Human IgG-ɣ-HRP conjugate diluted 1/10,000 in dilution buffer. After washing the plates 5 times in washing buffer, TMB (3,3’,5,5’-tetramethylbenzidine) substrate (KPL) was added and plates incubated for 30 min at room temperature. Reaction was stopped by addition of 1 M H_3_PO_4_, and absorbance read at 450 nm. A qualitative competition assay was carried out using a similar protocol, but pre-incubating the samples with either free S-(–)-nicotine (1 μM) or S-(–)-cotinine (50 μM) as competitors prior to addition to the coated and blocked ELISA plates. Candidates displaying ≤60% competition with S-(–)-nicotine or >20% competition with cotinine were eliminated. This step was included to ensure elimination of hits specific to pGlu or an epitope covering atoms at the interface of S-(–)-nicotine, pGlu and the amide bond connecting both; as well as candidates cross-reacting with cotinine.

#### Nicotine assay

Blood and brain were collected as described within each experiment. Trunk blood or venous blood were centrifuged at 2,000 x *g* to obtain serum. Brains were rinsed in water and both serum and brains were stored at -20 °C until measured via gas chromatography. Nicotine concentrations in serum or brain were measured using gas chromatography with nitrogen-phosphorus detection [34,35]. Concentrations that were below the 2 ng/mL limit of quantitation for the assay were treated as being 1 ng/mL for the purposes of analysis. Brain nicotine levels were corrected for brain-blood content as previously described [34].

#### Control mouse monoclonal antibody NIC9D9 preparation and in vitro characterization

The murine nic•mAb NIC9D9 (licensed from The Scripps Research Institute, La Jolla, CA by Antidote Therapeutics, Inc.) was selected as a reference antibody and produced from a hybridoma cell line [36,37]. This nic•mAb has been previously characterized with high specificity for S-(–)-nicotine [38]. The clarified and filtered NIC9D9-containing supernatant was concentrated, and buffer exchanged by tangential flow filtration, and the solution titrated to pH 5.0 with acetic acid. The material was loaded onto a 20 mL Capto S cation exchange column (GE Life Sciences), and the antibody eluted in 1–2 column volumes of 200 mM sodium acetate, pH 5.5. Relevant fractions were pooled and dialyzed against a 200-fold volume of 1x PBS, and purified antibody concentrated to 10 mg/mL using Amicon Ultra centrifugal filter units (Millipore). Purity was >90% by SDS-PAGE, and functional quality control assays were conducted analyzing binding to a S-(–)-nicotine-BSA conjugate in ELISA format (essentially as described in Isomura et al. 2001 [37]).

#### Affinity of monoclonal antibodies for nicotine

The affinity for free S-(–)-nicotine in solution of the Nic•mAb candidates displaying >60% competition with S-(–)-nicotine or ≤20% competition with S-(–)-cotinine was determined using the BiOptix 404pi enhanced SPR instrument. Antibodies were immobilized on a BiOptix CMD200m sensor chip using standard EDC/NHS amine coupling with blocking of the remaining active esters with ethanolamine. S-(–)-nicotine was injected at different concentrations (3x serial dilutions) in running buffer (10 mM HEPES (pH 7.4), 150 mM NaCl, 3 mM EDTA, 0.05% Tween-20), followed by buffer flow at 25°C to monitor dissociation. *K*_D_ values were calculated based on the on- and off-rate constants using the Scrubber2 software (BioLogic Software, Campbell, Australia).

To confirm the findings using SPR, a solution binding experiment was performed by equilibrium dialysis using ^3^H-nicotine on microtiter plates as previously described [39]. Briefly, duplicate samples of a given nic•mAb were diluted to a concentration of approximately 2.8 μg/mL and added to one side of the membrane in 96-well equilibrium dialysis plates. The dialysate side consisted of a range of nicotine concentrations (8–256 ng/mL) mixed with approximately 10^4^ DPM/0.1 mL radiolabeled nicotine. Plates were placed on a shaker for 72 h at room temperature. The radiolabeled nicotine was used to measure nicotine concentrations on both sides of the membrane. Nicotine binding affinity (represented by the *K*_D_) was measured from One-site–Specific Binding using Prism 8.0 (GraphPad Software Inc., La Jolla CA).

#### Antibody selectivity

The selectivity of the nic•mAbs 5G4, 7A8, 12F5, and 8D1 for S-(–)-nicotine was determined by competitive ELISA using 3’AM-S-(–)-Nic-pGlu-coated plates essentially as described above. Serial dilutions (in a blocking/diluent buffer of PBS + 1% non-fat dry milk) of competitors were added to the coated and blocked plates. Subsequently, the nic•mAb was added at a fixed concentration yielding a non-saturating A_450_ level in control wells without competitor. After 45 min incubation at 37°C, the plates were washed, and detection of bound nic•mAb was done as previously described. Small molecule competitors included S-(–)-nicotine, acetylcholine chloride, β-nicotinamide adenine dinucleotide (NAD), bupropion, S-(–)-cotinine, cytisine, dopamine (3-hydroxytyramine) hydrochloride, mecamylamine hydrochloride, nicotinamide, (±)-norepinephrine (+)-bitartrate, (±)-nornicotine, serotonin hydrochloride, and varenicline tartrate (all from Sigma). These compounds include neurotransmitters, nicotine metabolites, structurally similar compounds, and smoking cessation drugs. The cross-reactivity was calculated from the ratio of the IC_50_ values as follows: (IC_50_, nicotine/IC_50_, competitor) × 100%.

#### Fully human Nic•mAb preparation

To reduce/eliminate Fc effector function (complement-dependent cytotoxicity or antibody-dependent cellular cytotoxicity), which is not needed for binding and neutralizing nicotine and could be a liability in the event of cross-reactivity with membrane targets, 5G4, 7A8, 12F5, and 8D1 were reformatted from the IgG1 to the IgG4 isotype [40] and named ATI-1010 through ATI-1013, respectively. Additional Fc sequence modifications were concurrently made to prevent Fab-arm exchange *in vivo* [41]. The fully human nic•mAbs ATI-1010, 1011, 1012, and 1013 were produced in sufficient quantity for *in vivo* testing using the ExpiCHO Expression System (Thermo-Fisher). Essentially, ExpiCHO cells were expanded at 37°C in 2L shake flasks. Cultures were adjusted to 6x10^6^ cells/mL and transfected per the vendor-supplied protocol. Cultures were fed on days 1 and 5 post-transfection, and the temperature was lowered to 32°C on day 1. Cultures were harvested on day 12 by centrifugation, 0.22 μm filtered, and stored at 4°C. Purification was performed on a Protein A affinity column (MAbSelect resin (GE Life Sciences)) with glycine pH 3.0 elution. After neutralization and pooling of the main peak fractions, the material was exchanged into PBS by dialysis. Purity was ≥95% by SDS-PAGE with Coomassie blue staining.

#### Stable cell pool generation and expression for production of ATI-1013

For the larger quantities of ATI-1013 required for conducting the *in vivo* addiction and non-GLP toxicology studies, stable-pools of ATI-1013 were generated in CHO-DG44 cells (Aragen Bioservices). Following transfection by electroporation and selection for stable transfectants, mini-pools were generated and gene amplification was conducted using gradually increasing methotrexate (MTX) and G418 (Geneticin) selection over 3 weeks in a stepwise manner. The top 120 mini-pools were expanded and evaluated with normalized cell densities in a 24-well format to obtain a more accurate ranking. The 20 mini-pools with the highest titers from the 24-well plates were evaluated in shake flasks (50 mL Gibco CD OptiCHO + 8 mM L-glutamine; ThermoFisher Scientific), and mini-pool 117 gave the highest titer. SDS-PAGE resulted in expected band sizes. Further scale-up to 1.4L shake flask reached a peak cell density (18 x 10^6^ cells/ml) at day 11 with harvest at day 14 with 65% viability yielding 1.3 g/L titer at harvest. Mini-pool 117 was then scaled-up further to 16L in a WAVE^™^ bioreactor (GE Life Sciences). Purification was conducted using Protein A affinity purification (MabSelect SuRe, GE Life Sciences), with elution in 0.1 M Glycine, pH 3.0, and neutralization using 1.0 M Tris, pH 8.0, followed by flow-through anion exchange chromatography (CaptoQ; GE Life Sciences). Concentration and buffer exchange into formulation buffer (25 mM L-Histidine, 250 mM Sucrose, pH 7.0) was performed using tangential flow filtration (Sartorius Vivaflow 200 30kDa). Final purity was >95% by SDS-PAGE with a monomeric purity of >99% as estimated by SEC.

#### Experiment 1: Effect of Nic•mAb on distribution of a single nicotine dose

Adult female SD rats (n = 8/group) were obtained with a jugular venous catheter in place. ATI-1010, 1011, 1012, and 1013 were dosed at 20 mg/kg i.p. 2 h prior to i.v. dosing of 0.03 mg/kg nicotine via jugular catheter. NIC9D9, a mouse monoclonal antibody with high affinity for nicotine (*K*_D_ = 43 nM) [38] and known *in vivo* activity, was included as a positive control. PBS alone (vehicle) was administered 1 mL/kg as a negative control. Blood and brains were collected 3 min after drug administration, processed, and assayed as described above (see Nicotine assay).

#### Experiment 2: Effect of escalating Nic•mAb doses on distribution of a single nicotine dose

To investigate dose effects of Nic•mAb on distribution of nicotine, an experiment identical to *Experiment 1* was performed using age-matched groups of male and female adult SD rats (n = 8/group, 4M and 4F per group) except that rats received vehicle, 10, 20, or 40 mg/kg i.p. of ATI-1012 and ATI-1013 2 h prior to administration of 0.03 mg/kg nicotine i.v.. Rats that had less than 5 μg/mL serum antibody levels (indicating incomplete nic•mAb administration due to catheter malfunction), as measured by ELISA, were excluded from the analyses. The excluded animals had an average serum antibody level of 0.73 μg/mL. The number of excluded animals are as follows for ATI-1012 administration (parentheses indicate the ATI-1012 mg/kg dose): 2 (10), 2 (20), and 4 (40); and for ATI-1013 administration (parentheses indicate the ATI-1013 mg/kg dose): 2 (10), 3 (20), and 2 (40).

#### Experiment 3: Estimation of Nic•mAb pharmacokinetic parameters

Adult female SD rats (n = 3/group) were obtained with a jugular venous catheter in place (Charles River, PA). Rats received 5 mg/kg i.v. ATI-1012 or ATI-1013 via tail vein. Blood (0.4 mL) was collected into serum separator tubes via the jugular catheter at pre-dose, at 10 and 30 min, and at 2, 6, 12, 24, 48, 72, and 120 h. Serum was isolated and stored at -20°C until analysis. The serum levels of ATI-1012 or ATI-1013 were determined using a concentration-based ELISA and quantitated against serial dilutions of the dosed mAb.

#### Experiment 4: Effect of Nic•mAb on distribution of repeated nicotine doses

Adult HSD rats were anesthetized with 0.1 mg/kg fentanyl, 0.05 mg/kg dexmedetomidine i.m., and 100 mg/kg propofol i.p., and a jugular venous catheter was placed. Two groups of rats (n = 10/group, 5M and 5F per group) received ATI-1013 40 or 80 mg/kg i.v. via jugular catheter and one group received 80 mg/kg of non-specific human polyclonal IgG (Gammagard; Baxter Healthcare Corp., Westlake Village, CA) as a control. Twenty-four hours later, 0.03 mg/kg nicotine was dosed i.v. once every 10 min via jugular catheter for a total of 5 doses. Blood was sampled 3 min after the first dose of nicotine. Animals were sacrificed 3 min after the fifth nicotine dose, i.e., 43 min after the first nicotine dose and blood and brain collected. Three rats (1 female that received 40 mg/kg ATI-1013 and 1 female and 1 male that received 80 mg/kg ATI-1013) had high brain nicotine levels, suggesting contamination during measurement, were removed from analyses. Outliers were identified using ROUT (Q = 1%).

#### Experiment 5: Effect of Nic•mAb on self-administration of nicotine

Adult male SD rats were initially trained for nicotine self-administration (NSA) using a unit nicotine dose of 0.03 mg/kg under a fixed-ratio (FR) 3 schedule during 2 h sessions using our routine protocol [42,43]. This unit dose is a common training dose for nicotine self-administration and was chosen to facilitate acquisition [31]. Sessions were conducted five days per week (Monday through Friday). After stable NSA was established (at least 5 infusions per session, an active:inactive lever ratio of at least 2:1, and no trend in the number of infusions over five consecutive sessions), the unit dose was reduced to 0.015 mg/kg, which results in serum nicotine concentrations during 2 h sessions that are more similar to smoking in humans [44,45]. After NSA stabilized at this unit dose (same criteria as above) on a Friday, rats (n = 7/group) began receiving twice-weekly i.v. infusions of 160 mg/kg ATI-1013 or 160 mg/kg Gammagard (control mAb) 30 min prior to the session on the following Mondays and Thursdays while rats continued NSA at the 0.015 mg/kg dose for 10 consecutive sessions (2 weeks). Then, the unit nicotine dose was reduced to 0.0075 mg/kg on a Monday for another 10 consecutive sessions (2 weeks) while the twice-weekly nic•mAb treatment continued. A higher dose of ATI-1013 was chosen for this experiment because the self-administered cumulative nicotine dose was higher (over twice in some rats) than the experimenter-administered nicotine doses in the other experiments.

#### Experiment 6: ATI-1013 repeat dose pharmacokinetics experiment

Age-matched adult SD rats (n = 6/group, 3M and 3F per group) were obtained with femoral vein and jugular vein catheters in place (FVC and JVC, respectively; Charles River, PA) received i.v. doses via the FVC of 40 mg/kg ATI-1013 weekly for 4 weeks. Residual nic•mAb concentrations were measured in serum from blood draws via the JVC at various time points after i.v. dosing. The ELISA detection assay employed relies on nicotine conjugate binding to 3’AM-S-(–)-Nic-pGlu [29] and thus reflects functional nic•mAb levels binding S-(–)-nicotine in serum. The first 4 time-points after ATI-1013 administration (but before the second dose was given) were used to measure ATI-1013 half-life to validate the findings from Experiment 3. At the end of this study, rats were dosed with 0.03 mg/kg i.v. nicotine and sacrificed 3 min later and samples were analyzed to assess the amount of unbound nicotine before and after ultrafiltration [46].

#### Experiment 7: Toxicology assessment of high doses of ATI-1013

The purpose of this study was to evaluate the repeat-dose tolerability of four (4) fixed i.v. doses of 200 mg/kg ATI-1013 dosed once weekly in the presence of 1 mg/kg/day of nicotine given continuously by s.c. for 28 days at Noble Life Sciences, beginning on study day 1.

Weight matched (225-300g) female and male SD rats were divided into four groups (n = 16/group, 8M and 8F per group). Animals were implanted with osmotic pumps delivering a continuous dose of nicotine or vehicle through the study period. ATI-1013 was delivered weekly by intravenous injection. Group 1 received saline, group 2 received nicotine only, group 3 received ATI-1013, and group 4 received ATI-1013 and nicotine.

Body weights were collected twice weekly and cage-side observations made daily. On study day 29, blood was collected via the jugular vein under anesthesia and analyzed for hematology, serum chemistry, and coagulation. Additional aliquots of blood were taken for assaying serum levels of ATI-1013. After blood collection animals were placed under anesthesia with isoflurane and sacrificed by thoracotomy. Tissues were harvested and weighed by tissue with kidneys, testis, and ovaries weighted as a pair.

Assessment of toxicity was based on mortality, clinical observations, and body weight during the 28 d study and at the end of study on organ weights, gross anatomic pathology, hematology, serum clinical chemistry, and coagulation. Tissue histopathology evaluations for heart, liver, lung, kidney, spleen, skeletal muscle, brain, colon, stomach, ovary, and testis have been conducted. Tissues were fixed immediately in formalin, embedding in paraffin, staining with H&E, and were reviewed by a veterinary pathologist.

#### Data analysis

In the single-dose nicotine pharmacokinetic studies serum or brain nicotine concentrations were compared by Welch’s one-way ANOVA with Dunnett’s T3 multiple comparisons test. Non-parametric Kruskal-Wallis testing was also conducted to assess the robustness of ANOVA’s conclusions based on parametric assumptions for the relatively small sample size used in these experiments. Estimates of pharmacokinetic parameters (volume of distribution, clearance, terminal half-life) were obtained from serum concentrations using noncompartmental analysis (PKSolver) [47]. In the multiple dose nicotine pharmacokinetic studies, serum and brain nicotine concentrations were compared between the different doses of ATI-1013 and IgG groups using two-tailed unpaired t-tests with Welch’s correction for unequal variances. For nicotine self-administration studies, the primary endpoint was the mean number of infusions during the last 3 sessions (Wednesday—Friday) at baseline and the last 3 sessions (Wednesday—Friday) at each unit dose during nic•mAb treatment. Because variances were unequal between groups, means were compared between groups using independent-samples t-tests with Welch’s correction and a significance level of p = 0.017 to account for the three comparisons. Mean infusions within each group between baseline and at each unit nicotine dose post-mAb administration were similarly analyzed but using paired t-tests.

## Results

### Novel monoclonal antibody leads

ELISA screening of 54 million single mAb expressing B-cells for the small subset that bound S-(–)-nicotine and did not bind S-(–)-cotinine (a main nicotine metabolite found at a 10- to 20-fold molar excess over nicotine in the blood of smokers, due to its significantly longer half-life) produced a set of 32 novel and unique, fully human anti-nicotine mAbs (nic•mAbs). Affinity testing using SPR showed that 10 of these mAbs had *K*_D_ values <70 nM for S-(–)-nicotine (Table 1).

**Table 1:** Affinity of the 10 most potent nicotine binding IgG1 mAbs and the name of their IgG4 isotype counterparts.

IgG1	k_on_ (M^-1^min^-1^)	k_off_ (min^-1^)	*K*_D_ (nM)^A^	IgG4
8D1	1.40x10^6^	0.0064	5	ATI-1013
12F5	8.40x10^5^	0.0171	20	ATI-1012
7A8	2.95x10^5^	0.0089	30	ATI-1011
5D1	4.18x10^5^	0.0127	30	
5G4	2.94x10^5^	0.0093	31	ATI-1010
5H1	1.31x10^6^	0.0467	37	
15A4	5.43x10^5^	0.0219	40	
13F7	3.53x10^5^	0.0190	54	
2E11	1.85x10^5^	0.0112	61	
8H5	9.64x10^5^	0.0637	67	

^A^Affinity measured by SPR; S-(–)-Nicotine; 25°C.

**Fig 1:**
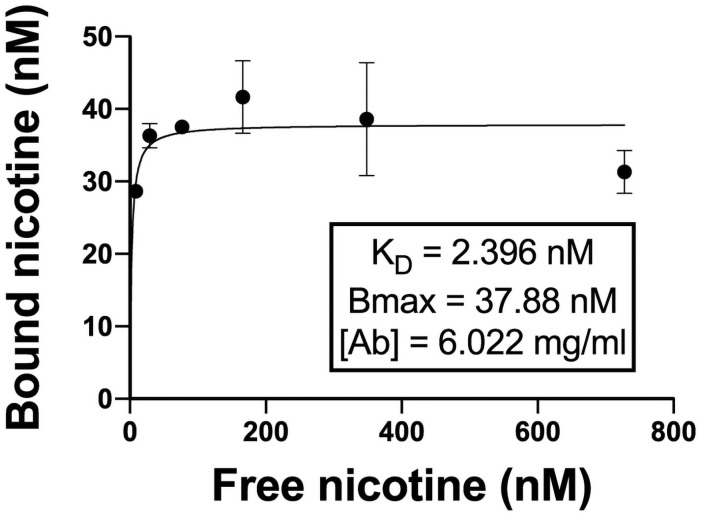
Measurement of ATI-1013 affinity for S-Nicotine. Affinity was measured by equilibrium dialysis at 25°C using ^3^H-nicotine. Duplicate samples of ATI-1013 were diluted to 2.8 μg/mL and plated to one side of 96-well equilibrium dialysis plates. The dialysate side consisted of a range of nicotine concentrations (8–256 ng/mL) mixed with approximately 10^4^ DPM/0.1 mL radiolabeled nicotine. Plates were placed on a shaker for 72 h at room temperature. The radiolabeled nicotine was used to measure nicotine concentrations on both sides of the membrane. *K*_D_ and B_max_ were measured using One-site–Specific Binding in Prism 8.0 (GraphPad Software Inc., La Jolla CA). Data are represented as mean ± SD.

The 4 highest affinity lead antibodies were denoted 5G4, 7A8, 12F5, and 8D1, and selected for additional analyses. Antibody 5G4 was selected over 5D1 due to 5G4’s higher affinity measured by SPR at 37°C (86 nM vs. 105 nM, respectively). 5G4, 7A8, 12F5, and 8D1 were reformatted from IgG1 to IgG4 isotypes and renamed to ATI-1010 through ATI-1013, respectively. To confirm the findings using SPR and that the reformatting to the IgG4 isotype did not negatively impact binding affinity, the affinity of ATI-1013 for S-(–)-nicotine was measured by equilibrium dialysis (Fig 1). The *K*_D_ was 2.4 nM at 25°C, consistent with the value of 5 nM obtained by SPR at 25°C for 8D1 (Table 1). The expression of ATI-1010 through ATI-1013 was scaled up in a transient ExpiCHO expression system for *in vivo* testing. ATI-1012 and ATI-1013 were purified to >95% purity (assessed by SDS-PAGE) with >95% monomer content measured by analytical SEC using a standard protein A purification protocol, whereas ATI-1010 and ATI-1011 exhibited high molecular weight species and/or aggregate formation (<90% monomer content by SEC).

The 4 highest affinity leads, ATI-1010, 1011, 1012, 1013, were then evaluated for selectivity against a panel of relevant compounds including neurotransmitters, nicotine metabolites, structurally similar compounds, and smoking cessation drugs. ATI-1012 and 1013 exhibited the highest selectivity for S-(–)-nicotine over the compounds in the panel (Table 2). For both leads, no binding was detected (>10^6^-fold selectivity for nicotine) to cotinine, acetylcholine, nicotinamide, serotonin, bupropion, mecamylamine, and nornicotine. ATI-1012 only had nominal binding to norepinephrine (~3x10^3^-fold selectivity). ATI-1013 had negligible binding (≥ 5x10^6^-fold selectivity) to NAD, varenicline, dopamine, norepinephrine, and cytisine.

**Table 2:** Antigen selectivity of the 4 nic•mAbs^A^ with highest affinity for S-(–)-nicotine.

Compound	Cross-reactivity (%)^B^			
	1013	1012	1011	1010
S-(–)-nicotine	100	100	100	100
S-(–)-cotinine	NCR	NCR	0.094	0.035
Acetylcholine chloride	NCR	NCR	NCR	NCR
Nicotinamide	NCR	NCR	NCR	0.0004
Dopamine HCl^C^	0.0003	NCR	NCR	NCR
Serotonin HCl	NCR	NCR	NCR	NCR
(+/-)-Norepinephrine (+)-bitartrate salt	0.0005	0.030	0.0020	0.0015
Nornicotine	NCR	NCR	0.056	0.20
Bupropion	NCR	NCR	NCR	0.011
Cytisine	0.0001	NCR	NCR	NCR
Varenicline tartrate	0.0002	NCR	NCR	0.0018
β-Nicotinamide adenine dinucleotide (NAD)	0.0002	NCR	0.0036	0.0037
Mecamylamine HCl	NCR	NCR	NCR	NCR

NCR, no cross-reactivity.

^A^ IgG4-isotype.

^B^ (IC_50_ of Nicotine/IC_50_ of Compound) x 100%.

^C^ 3-Hydroxytyramine HCl.

### Experiment 1: Effect of nic•mAb on distribution of a single nicotine dose

**Fig 2:**
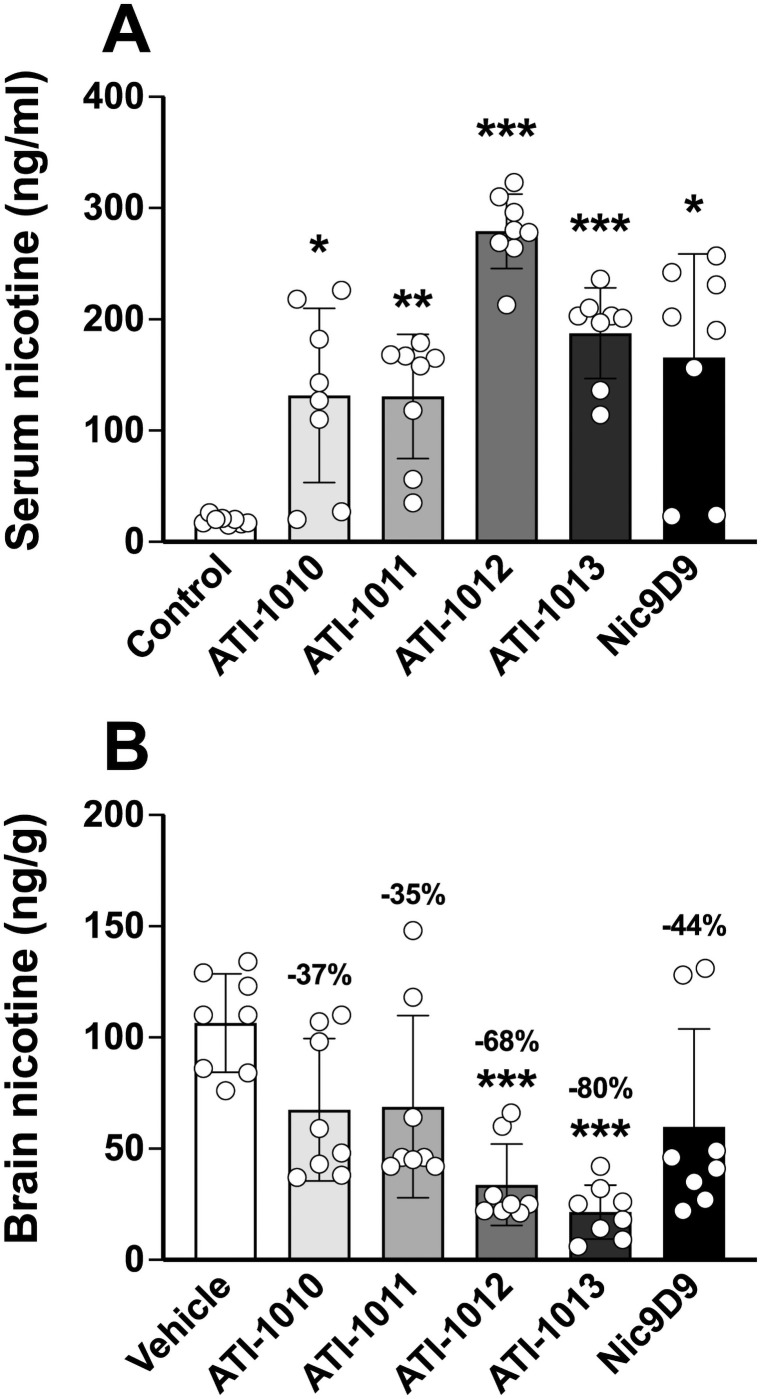
Functional *in vivo* activity of the 4 nic•mAbs with highest affinity for nicotine. Rats (n = 8/group) were pretreated i.p. with 20 mg/kg ATI-1010, 1011, 1012, 1013, NIC9D9, or PBS alone (vehicle) 2 h prior to i.v. dosing of 0.03 mg/kg nicotine via jugular catheter. Blood and brains were collected 3 min after drug administration. Data represent mean serum (Panel A) and brain (Panel B) nicotine concentrations in each group. *p<0.05, **p<0.01, ***p<0.001 significantly different from vehicle. Numbers above bars represent the percent difference from controls. One-way ANOVA with Dunnett’s T3 multiple comparisons test was used to compare groups to vehicle. Data are expressed as mean ± SD.

All nic•mAbs significantly increased serum nicotine levels compared to controls 3 min after a 0.03 mg/kg i.v. nicotine dose (Fig 2A). Only ATI-1012 and 1013 had significantly lower brain nicotine levels compared to control (68% and 80% lower, respectively) (Fig 2B).

### Experiment 2: Effect of escalating nic•mAb doses on distribution of a single nicotine dose

**Fig 3:**
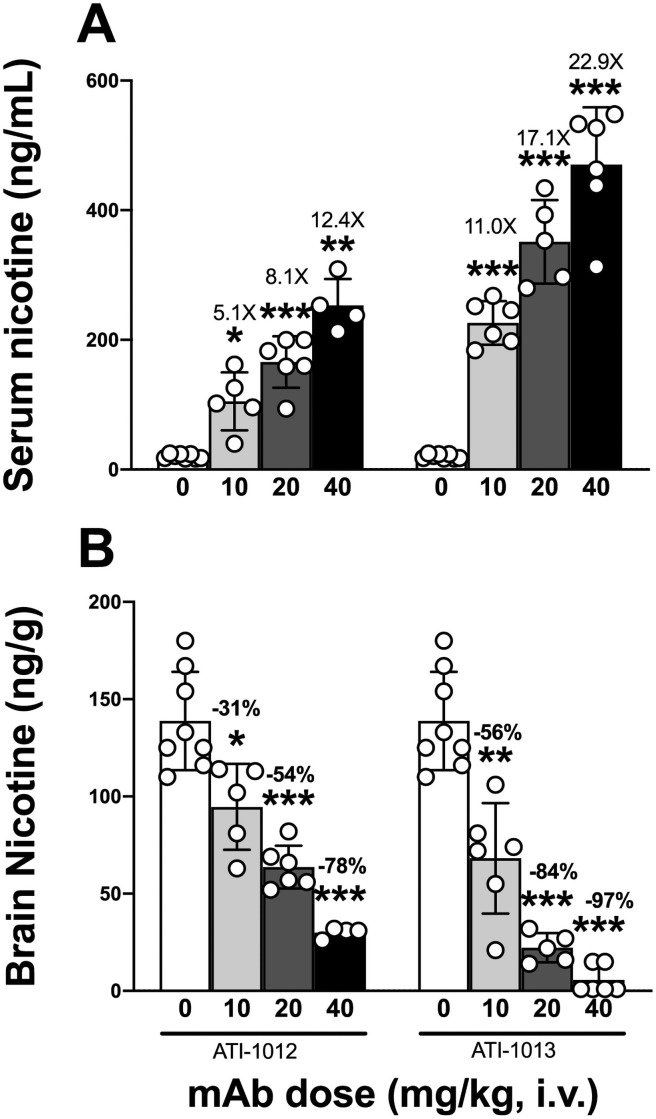
Single dose nicotine pharmacokinetics in rats pre-treated with ATI-1012 or ATI-1013. Doses of 10, 20, and 40 mg/kg ATI-1012, ATI-1013 and control (0 mg/kg) were given to rats (n = 8/group), followed by administration of 0.03 mg/kg nicotine i.v. in <10 s. Animals were sacrificed after 3 min and nicotine was measured in serum (Panel A) and brain (Panel B). Numbers above the bars indicate fold increase in serum nicotine or the percentage remaining in brain. Due to patency issues, some animals did not receive adequate nic•mAb (refer to the Methods section for more details). *p<0.05, **p<0.01, ***p<0.001 significantly different compared to controls. Data are expressed as mean ± SD.

Based on the results that ATI-1012 and ATI-1013 significantly lowered brain nicotine levels compared to controls and were more potent than ATI-1010 and ATI-1011 at each dose tested (along with the observation that ATI-1010 and 1011 showed high molecular aggregates upon expression in ExpiCHO), varying doses of ATI-1012 and 1013 were evaluated in a second single-dose nicotine-pharmacokinetic study. Pre-treatment with ATI-1012 and 1013 increased serum nicotine levels and reduced nicotine brain levels in rats given a single i.v. dose of 0.03 mg/kg nicotine, equivalent to the nicotine delivered by smoking 2 cigarettes (Fig 3).

Rats pretreated with vehicle, 10, 20, and 40 mg/kg ATI-1012 produced mean nicotine serum levels of 21, 105, 166, and 253 ng/mL 3 min following a 0.03 mg/kg i.v. nicotine dose, which corresponded to a 5, 8, and 12-fold increase over the vehicle treated group, respectively (Fig 3A). There was a significant increase of nicotine in serum in all ATI-1012 groups compared to controls. Mean brain nicotine levels in vehicle, 10, 20, and 40 mg/kg ATI-1012 groups were 139, 95, 64, and 30 ng/g, which corresponded to a 31%, 54%, and 78% reduction compared to the vehicle treated group, respectively (Fig 3B). There was a significant decrease in nicotine concentration in brain in all ATI-1012 groups compared to controls. Two-way ANOVA indicated statistically significant difference by dose and treatment (both p<0.0001). Dunnett’s test for multiple comparisons indicated that each individual dose was significantly different than controls.

Rats pretreated with vehicle, 10, 20, and 40 mg/kg ATI-1013 produced mean nicotine serum levels of 21, 226, 351, and 470 ng/mL 3 min following a 0.03 mg/kg s.c. nicotine dose, which corresponded to an 11, 17, and 22-fold increase over the vehicle treated group, respectively (Fig 3A). There was a significant increase of nicotine in serum in all ATI-1013 groups compared to controls. Mean brain nicotine levels in vehicle, 10, 20, and 40 mg/kg ATI-1013 groups were 139, 68, 22, and 4 ng/g, which corresponded to a 56%, 84%, and 97% reduction compared to the vehicle treated group, respectively (Fig 3B). There was a significant decrease in nicotine concentration in brain in all ATI-1013 groups compared to controls. Two-way ANOVA indicated statistically significant differences by dose and treatment (both p<0.0001). Dunnett’s test for multiple comparisons indicated that each individual dose was significantly different than controls.

### Experiment 3: Estimation of nic•mAb pharmacokinetic parameters

**Fig 4:**
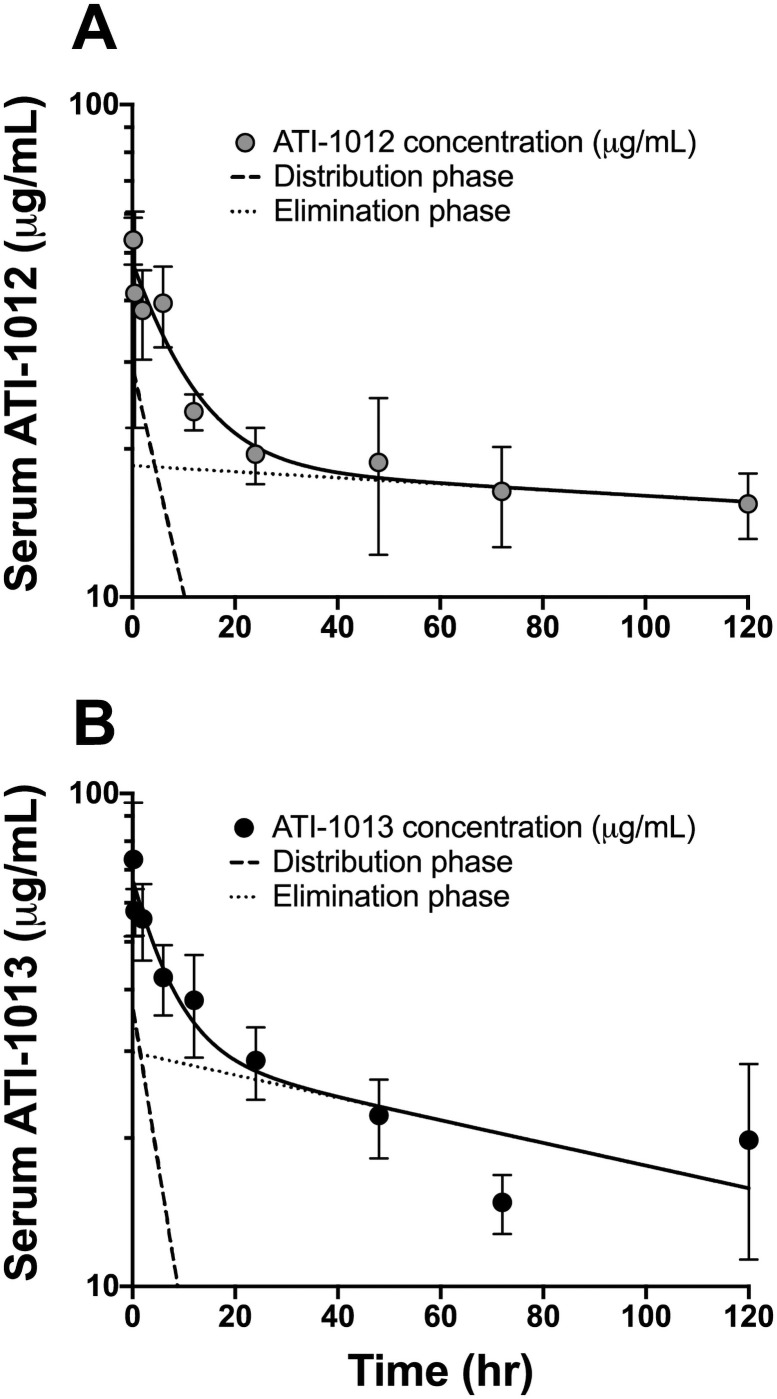
Pharmacokinetics of ATI-1012 and ATI-1013 in rats. Female SD rats were obtained with a jugular venous catheter in place. Rats (n = 3/group) received 5 mg/kg i.v. ATI-1012 (Panel A) or ATI-1013 (Panel B) via tail vein. Blood (0.4 mL) was collected into serum separator tubes via the jugular catheter at pre-dose, at 10 and 30 min, and at 2, 6, 12, 24, 48, 72, and 120 h. Noncompartmental analysis (PKSolver) of ATI-1012 and ATI-1013 showed elimination half-lives of 493 h and 131 h, respectively. Data are expressed as mean ± SD.

The PK properties of the two lead candidates ATI-1012 and ATI-1013 were explored in rats. Residual nic•mAb concentrations were measured at various time points after i.v. dosing (Fig 4). The detection assay employed relies on nicotine conjugate binding, and thus reflects functional nic•mAb levels in serum. Parameters estimated by noncompartmental analysis of ATI-1012 concentrations include an elimination phase half-life of 493 h, clearance of 0.10 mL/min/kg, and a steady-state volume of distribution of 0.15 L/kg (Fig 4A). The corresponding values for ATI-1013 were 131 h, 0.10 mL/min/kg, and 0.08 L/kg, respectively (Fig 4B).

### Selection of ATI-1013 as the primary lead candidate

While ATI-1012 and ATI-1013 both had acceptable selectivity profiles, *in vivo* circulating half-lives, efficient expression and good purification results, ATI-1013 had greater functional activity *in vivo* as compared to ATI-1012 at each dose tested as well as comparable selectivity for S-(–)-nicotine. Thus, ATI-1013 was selected as the definitive lead antibody and ATI-1012 reserved as a back-up compound. ATI-1013 was then progressed for further qualification by repeat-dose nicotine experiments simulating very heavy smoking and behavioral addiction studies *in vivo* as described below.

### Experiment 4: Effect of nic•mAb on distribution of repeated nicotine doses

**Fig 5:**
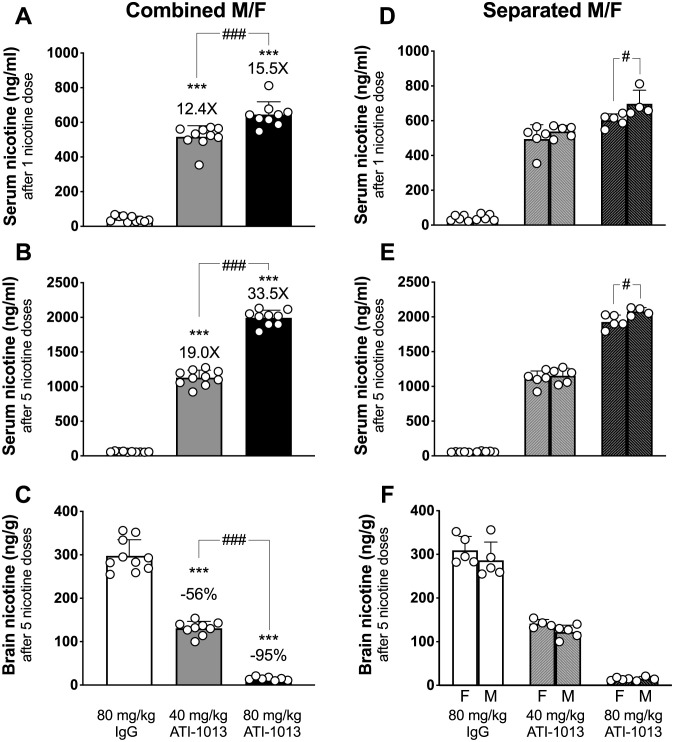
ATI-1013 alters nicotine distribution to serum and brain following repeated administration of nicotine. Two groups of rats (n = 10/group) received ATI-1013 40 or 80 mg/kg i.v. via jugular catheter and one group received 80 mg/kg of non-specific IgG as a control. Twenty-four hours later, 0.03 mg/kg nicotine was dosed i.v. once every 10 min via jugular catheter for a total of 5 doses. Blood was sampled 3 min after the first dose of nicotine (Panels A & D). Animals were sacrificed 3 min after the fifth nicotine dose, i.e., 43 min after the first nicotine dose and blood and brain collected (Panels B, C, E & F). Panels D, E &F show a comparison of results grouped by gender (M, male; F, female). ***p<0.001 significantly different compared to controls. ^#^p<0.05 and ^###^p<0.001 significantly different compared to designated group. Data are expressed as Mean ± SD.

To test the effects of ATI-1013 in a simulated scenario of acute heavy smoking, rats pre-treated with ATI-1013 or control IgG received a series of 5 repeated i.v. nicotine doses spaced 10 min apart. Total serum nicotine increased as a function of accumulated nicotine dosing, and in an ATI-1013 dose-dependent manner after both the first and fifth nicotine dose (Fig 5). Serum nicotine levels in 40 mg/kg and 80 mg/kg ATI-1013 treated rats were increased by 12.4- and 15.5-fold, respectively, compared to controls following the first nicotine dose and increased by 19.0- and 33.5-fold, respectively, following the fifth nicotine dose. Brain nicotine levels in 40 mg/kg and 80 mg/kg ATI-1013 treated rats were decreased by 56% and 95%, respectively, compared to controls following the fifth nicotine dose (Fig 5C).

Serum nicotine levels were significantly different between males and females in the group treated with 80 mg/kg ATI-1013 following both the first and fifth nicotine doses (Fig 5D and 5E). Brain nicotine levels were not significantly different between males and females in any group (Fig 5F).

### Experiment 5: Effect of nic•mAb on self-administration of nicotine

**Fig 6:**
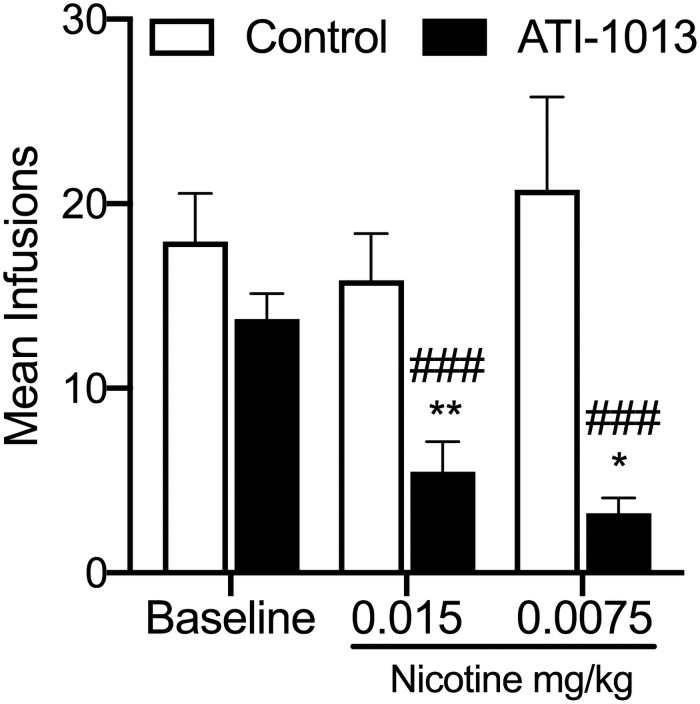
Nicotine self-administration in rats treated with ATI-1013. Mean infusions (n = 7/group) during the last three sessions of baseline (0.015 mg/kg unit dose, prior to nic•mAb treatment) and the last three sessions at each unit nicotine dose during twice-weekly (Monday and Thursdays) nic•mAb treatment. Sessions were 2 h in duration. Open bars represent data from rats treated with the nonspecific mAb Gammagard (160 mg/kg i.v.). Closed bars represent data from rats treated with ATI-1013 (160 mg/kg i.v.). ^##^p<0.01 and ^###^p<0.001 significantly different from baseline. *p<0.05 and **p<001 significantly different from control. Data are expressed as mean ± SEM.

Fig 6 shows the mean (± SEM) number of nicotine infusions during the last three sessions before (Baseline) and during nic•mAbs treatment at each unit nicotine dose. Rats given ATI-1013 exhibited a significant decrease in NSA at both nicotine unit doses compared to their respective baseline (p<0.001 at both doses) and compared to control rats (p<0.05 at both doses).

### Experiment 6: ATI-1013 repeat dose pharmacokinetics experiment

**Fig 7:**
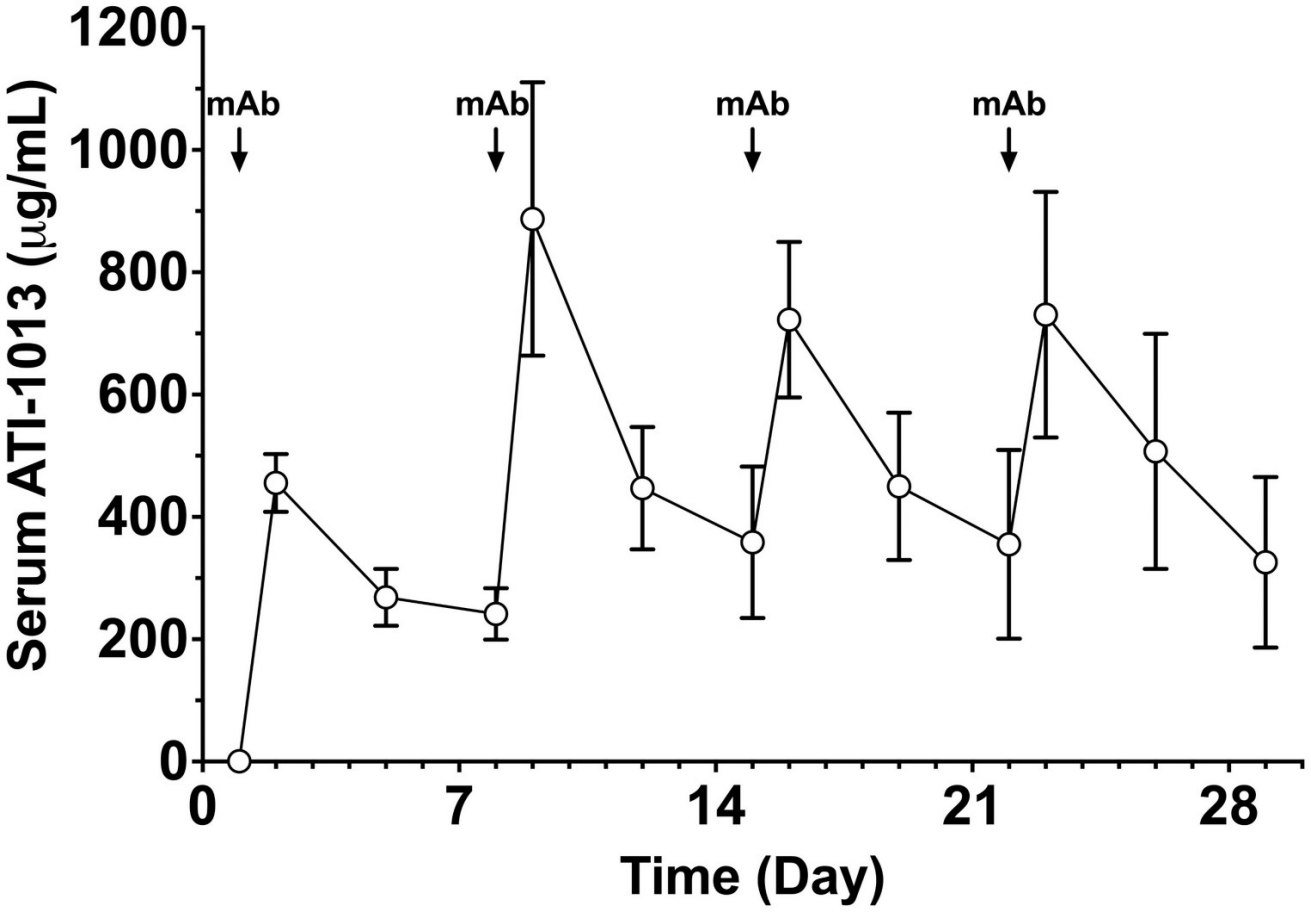
Distribution of ATI-1013 after weekly repeated dosing in rats. Age-matched SD rats (n = 6, 3M and 3F) received i.v. doses via FVC of 40 mg/kg ATI-1013 weekly for 4 weeks. Residual nic•mAb concentrations were measured at various time points after i.v. dosing. Arrows indicate times when ATI-1013 was administered. Data are expressed as mean ± SD.

Results demonstrated that high levels of ATI-1013 (> 200 μg/mL) could be maintained over several weeks (Fig 7). ATI-1013 mean serum concentrations of 456 and 887 μg/mL of ATI-1013 were achieved 24 h after the first and second dose, respectively (Fig 7). Mean trough serum levels 7 days after the first dose were 241 μg/ml and were 359 μg/ml 7 days following the last of the 4 weekly doses, suggesting some accumulation, although not statistically different. ATI-1013 half-life following administration of the first nic•mAbs dose was 176 ± 71.6 h estimated by noncompartmental analysis. At the end of the study, unbound nicotine was 2% 3 min following administration of 0.03 mg/kg nicotine.

### Experiment 7: Toxicology assessment of high doses of ATI-1013

**Fig 8:**
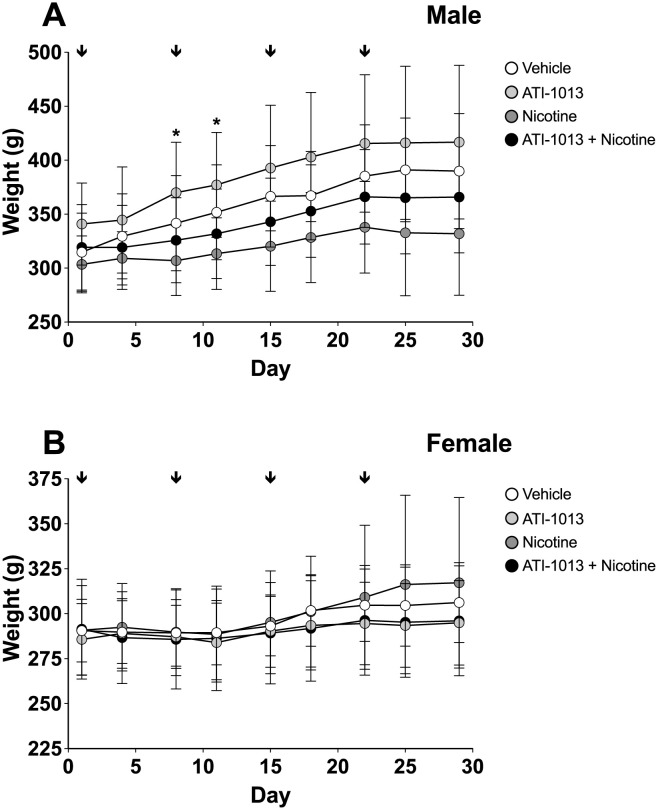
Body weight over time in toxicology study. Male (n = 8/group, Panel A) and female (n = 8/group, Panel B) SD rats were divided into four groups. Animals were implanted with osmotic pumps delivering a continuous dose of nicotine (1 mg/kg/day) or vehicle throughout the study period. ATI-1013 (200 mg/kg, i.v.) was delivered weekly. Group 1 received saline (open circles), group 2 received nicotine only (dark grey circles), group 3 received ATI-1013 (light grey circles), and group 4 received ATI-1013 and nicotine (closed circles). Arrows indicate times when ATI-1013 was administered. Mean ± SD. *p < 0.05 ATI-1013 group compared to nicotine alone group.

ATI-1013 was well-tolerated with no observed pathology in the treatment groups. All animals received the full dose with no injection related behavioral changes, injection site reaction, and no mortality was induced in any animals. Daily clinical observations found no observable behavioral changes or modifications in feeding or grooming in any groups. Body weight was monitored twice weekly for the duration of the study. Two-way ANOVA showed that nicotine treated male rats had lower body weight than ATI-1013 treated male rats on study days 8 and 11 (p<0.05) (Fig 8). No other significant differences between treatment groups were found. At the end of the study animals were necropsied and major organs (liver, lung, spleen, heart, kidneys, testis or ovaries) were isolated and weighed. No gross pathological findings were noted in these tissues and no statistically significant changes in organ weights were found. Blood was collected, and complete blood count performed to determine any changes in hematological parameters. While 4 animals across three groups had values outside the normal range (i.e. slightly decreased lymphocytes or hemoglobin), no clinically significant changes or trends were found between any groups. Moderate polychromasia was found in 6 animals in the following groups: Vehicle n = 1/16 (6%), ATI-1013 alone n = 0/16 (0%), Nicotine alone n = 2/16 (12%), ATI-1013+nicotine n = 2/16 (12%). A total of 3% in non-nicotine treated animals and 12% in nicotine treated animals. Serum clinical chemistry of 23 different analytes and plasma coagulation measures did not find any notable changes between treatment groups. Histopathological examination of major organ systems by a veterinary pathologist found no test article related lesions in any tissue examined. Tissues were specifically examined for any evidence of an immune histopathologic reaction, and none were observed.

## Discussion

The key findings of this study were that the fully human anti-nicotine mAb, ATI-1013: 1) had a very high affinity and selectivity towards nicotine; 2) had a very long half-life in rodent models; 3) dose-dependently reduced nicotine distribution to brain following multiple doses of nicotine simulating very heavy smoking; 4) significantly reduced the reinforcing effects of nicotine in a self-administration model; and 5) was demonstrated to be safe in a non-GLP toxicology study. These data suggest that ATI-1013 is safe, long-lasting, and at a sufficient dose could be an effective treatment for tobacco use disorders.

Nic•mAbs with increasing affinity towards nicotine have been demonstrated to be more effective at reducing distribution of nicotine to brain [8]. ATI-1013 was selected from a series of unique human-derived mAbs for its very high affinity for S-(–)-nicotine (*K*_D_ = 2.4 nM by equilibrium dialysis), high selectivity for S-(–)-nicotine and low selectivity for nicotine metabolites, smoking-cessation drugs, and neurotransmitters, and its potency in sequestering nicotine in the blood and thereby reducing brain nicotine levels. The affinity of ATI-1013 is very high compared to other reported high-affinity nicotine-specific monoclonal antibodies, including Nic311 (*K*_D_ = 60 nM) and NIC9D9 (*K*_D_ = 43 nM) [8,38]. Importantly, while it is possible that very high affinity would lead to saturation and reduced efficacy [48,49], ATI-1013 was shown to be effective following 5 repeated nicotine doses given 10 min apart, simulating very heavy smoking. In addition, the affinity of nicotine for neuronal α_4_β_2_ nicotinic acetylcholine receptors range from 1 to 15 nM [50–52] depending on experimental preparation and source of receptor, therefore, we postulate that the 2 nM affinity of ATI-1013 for S-(–)-nicotine may be more effective at reducing nicotine binding to α_4_β_2_ nicotinic acetylcholine receptors in the brain. ATI-1013 also does not bind to S-(–)-cotinine, a nicotine metabolite which is found at a 10- to 20-fold molar excess over nicotine in the blood of smokers due to its long half-life. This lack of cross-reactivity with cotinine indicates that high serum levels of S-(–)-cotinine should not interfere with the efficacy of ATI-1013 *in vivo*.

The pharmacokinetics of ATI-1013 showed a circulating half-life of 131 h in rats, which is similar to the half-life of nonspecific IgG in rats [53]. This was also confirmed in Experiment 6, whereby ATI-1013 was estimated to have a half-life of 176 h.

While ATI-1012 appeared to have a longer elimination half-life, this does not necessarily suggest that it would have an improved performance over ATI-1013 in humans. Rodent PK assays of mAb’s are not predictive of PK in humans but are often used as a measure of “*in vivo* fitness” in the lead selection process. An abnormally fast antibody clearance can be a sign of problematic nonspecific interactions, so these assays are often conducted for early-stage development screening to eliminate clones with such high nonspecific disposition PK [54]. None of the candidates displayed abnormally fast clearance that would warrant their elimination from further investigation.

The reduction in nicotine self-administration in the present study is consistent with the reduction in brain nicotine levels produced by ATI-1013, as well as the findings of other studies showing that nic•mAb treatment can reduce the behavioral effects of nicotine and other drugs of abuse [36,55–57]. These findings demonstrate that ATI-1013 reduces the reinforcing effects of nicotine, suggesting it may be useful for smoking cessation in humans. However, the dose of ATI-1013 used in the NSA study (160 mg/kg) was large, in part because the entire daily dose of nicotine was administered over a 2 h session, which is a very different and more intense dosing pattern than is typical of smokers. Further, the effective dose of ATI-1013 may be much lower in a clinical trial for smoking cessation because people will be motivated to quit, in contrast to rats in the present study continuously consuming nicotine during treatment. As a point of reference, effective doses of varenicline are more than an order of magnitude lower in clinical trials for smoking cessation than those needed to attenuate nicotine self-administration in preclinical studies in rats [58–60].

While it is not known what the clinically effective ATI-1013 dose and regimen will be, many examples of antibody products with high repeated dosages have been approved by FDA for treatment of other disorders, including evolocumab (420 mg once monthly), ibalizumab-uiyk (2.0 g loading dose, then 800 mg every two weeks), idarucizumab (5 g, up to 10 g for a single dose), casirivimab and imdevimab cocktail (two single i.v. doses of 1.2 g each) and human polyclonal IgG (e.g. Gammaguard 300–600 mg/kg every 3 to 4 weeks or 21 to 42 g/month for a 70 kg adult with primary immunodeficiency). Additionally, if needed, smaller doses of antibodies administered more frequently can lead to accumulation in plasma [61,62]. If multi-gram amounts of ATI-1013 are needed to achieve clinical efficacy, it will be important to optimize manufacturing yields in order to lower the cost of goods (COGS). Robust expression of ATI-1013 in stably transfected CHO-DG44 cell pools has been attained yielding 1.3 g/L of purified nic•mAb at 16L scale. Given this starting point, further improvements in yield are expected due to 1) selection of a higher expressing single clone as the master cell-line and 2) upstream and downstream process optimization, which may lead to reasonable titers of >3 g/L [63]. Published COGS estimates for monoclonal antibodies range from $50–$100/g for processes with titers ≥2 g/L [64] and given the advent of biosimilars, COGS are predicted to continue to decrease to <$20/g [63]. Therefore, we anticipate that based on the expression efficiencies already attained, along with additional process optimization, and the economies of larger commercial scale production, COGS will not be prohibitive to development of mAbs for smoking cessation.

An initial non-GLP 4-week repeated, high-dose toxicology study of ATI-1013 (200 mg/kg weekly) with and without concurrent administration of nicotine in rats showed that ATI-1013 was well-tolerated with no observed pathology in the treatment groups supporting continued development of ATI-1013 from a safety perspective. This result is not unexpected as other human mAbs have shown good safety profiles [65,66]. Lower body weight in male rats given nicotine alone was unsurprising since nicotine suppresses weight gain [67]. Nicotine addiction is recognized as a chronic relapsing disorder, where other important factors play a role in addition to nicotine (e.g. sensory aspects of smoking, situational triggers, etc.). Repeated and sustained quit attempts using a variety of approaches is often necessary for success [68]. Therefore, ATI-1013 could be used as a new tool by physicians within an integrated treatment and relapse prevention plan that includes behavioral counseling and other pharmacotherapies to help address factors that immunotherapy does not target (e.g. co-administering varenicline or bupropion, which are not bound by ATI-1013, to reduce craving and withdrawal).
